# Targeting DNA repair with PNKP inhibition sensitizes radioresistant prostate cancer cells to high LET radiation

**DOI:** 10.1371/journal.pone.0190516

**Published:** 2018-01-10

**Authors:** Pallavi Srivastava, Asitikantha Sarma, Chandra Mohini Chaturvedi

**Affiliations:** 1 Department of Zoology, Banaras Hindu University, Varanasi, Uttar Pradesh, India; 2 Radiation Biology Laboratory, Inter University Accelerator Centre, New Delhi, India; Northwestern University Feinberg School of Medicine, UNITED STATES

## Abstract

High linear energy transfer (LET) radiation or heavy ion such as carbon ion radiation is used as a method for advanced radiotherapy in the treatment of cancer. It has many advantages over the conventional photon based radiotherapy using Co-60 gamma or high energy X-rays from a Linear Accelerator. However, charged particle therapy is very costly. One way to reduce the cost as well as irradiation effects on normal cells is to reduce the dose of radiation by enhancing the radiation sensitivity through the use of a radiomodulator. PNKP (polynucleotide kinase/phosphatase) is an enzyme which plays important role in the non-homologous end joining (NHEJ) DNA repair pathway. It is expected that inhibition of PNKP activity may enhance the efficacy of the charged particle irradiation in the radioresistant prostate cancer cell line PC-3. To test this hypothesis, we investigated cellular radiosensitivity by clonogenic cell survival assay in PC-3 cells.^12^Carbon ion beam of62 MeVenergy (equivalent 5.16 MeV/nucleon) and with an entrance LET of 287 kev/μm was used for the present study. Apoptotic parameters such as nuclear fragmentation and caspase-3 activity were measured by DAPI staining, nuclear ladder assay and colorimetric caspase-3method. Cell cycle arrest was determined by FACS analysis. Cell death was enhanced when carbon ion irradiation is combined with PNKPi (PNKP inhibitor) to treat cells as compared to that seen for PNKPi untreated cells. A low concentration (10μM) of PNKPi effectively radiosensitized the PC-3 cells in terms of reduction of dose in achieving the same survival fraction. PC-3 cells underwent significant apoptosis and cell cycle arrest too was enhanced at G2/M phase when carbon ion irradiation was combined with PNKPi treatment. Our findings suggest that combined treatment of carbon ion irradiation and PNKP inhibition could enhance cellular radiosensitivity in a radioresistant prostate cancer cell line PC-3. The synergistic effect of PNKPi and carbon ion irradiation could be used as a promising method for carbon-ion therapy in radioresistant cells.

## Background

Radioresistance is an obstacle in the successful treatment of cancer by low LET radiotherapy using gamma radiation or X-ray radiation [1–5]. In the last several decades, high LET radiotherapy demonstrated favorable results for many malignancies that do poorly with conventional radiotherapy [6, 7]. The unique physical and biological properties of high LET radiation make it theoretically possible to carry out hypofractionated radiotherapy using a significantly smaller number of fractions than those used in conventional radiotherapy and this has become one of the important reasons to opt for it [8]. It has many potential advantages over the low LET radiotherapy as it overcomes the radioresistance problem along with permitting dose escalation within the tumor which might result in a better tumor control [9]. It has the property of delivering the maximum dose at the end of the particle range and so it will spare the normal surrounding tissues which results in reduction of side effects. However, at the entry path, a little amount of dose deposition is always there along the track. Hence the normal tissue is irradiated albeit with a far lesser dose which might lead to fibrosis etc. If the particle fluence is reduced in order to achieve dose reduction, this problem of irradiation of normal tissue could also be reduced further. High LET radiation creates clustered damage to the DNA which is considered as most lethal form of DNA damage [10]. This lethality can be observed as an increased relative biological effectiveness [11]. It has a smaller oxygen enhancement ratio and reduced cell cycle related radiosensitivity [12–14].

Despite the efficacy of high LET radiation therapy, the high cost and the relapse rates [15–17] indicate the urgent requirement for novel radiosensitizing strategies. High LET radiation has significant biological advantages and in combination with radiomodulators it may result in further enhancement in the efficiency and less number of dose fractions of radiotherapy (hypofractionated radiotherapy) will be required thus contributing to reduction in treatment cost. An added advantage would thus be that it will decrease the occurrence of severe side effects in normal tissues such as fibrosis [18, 19].

Prostate cancer (PCa) is the most frequently diagnosed tumor in men, accounting alone for 29% of cancer incidence, and it is the second most common cause of death due to cancer in men after lung cancer [20]. Low LET radiation has been serving as necessary component of therapy for PCa patients but the radioresistance of prostate cancer cells makes its treatment with radiation alone not very effective. The radioresistance problem ultimately leads to the local relapse and progression to metastatic disease in almost one third of PCa patients [21]. In our study we have used the PC-3 cell line, which is radioresistant because of its defective p53 compounded with overexpressed Bcl-2, which makes it antiapoptotic and resistant to cell death [14, 22–25]. Some studies have suggested that the,effect of C-ion radiation is independent of the status of p53 and Bcl-2 [14,26]. Due to these properties, treatment of prostate cancer is undergoing an evolution, shifting to the use of heavy ion species. It was noticed that PC-3 cells showed higher initial DNA damage and persistent cell cycle arrest after exposure of heavy ion species [27]. Dose- and time-dependent gene expression alterations were also observed in prostate and colon cancer cells when exposed to carbon ion beam [28]. The use of a radiosensitizer in combination with carbon ion radiation may further enhance cell death significantly.

In recent years, level of interest has been increased to inhibit DNA-repair pathways as an approach to potentiate the radio-therapeutic cancer treatments [11, 29–30]. Inhibitors of several enzymes involved in the repair of DNA strand breaks works as radiosensitizers and are currently at various stages of the drug development process [31–33]. Lee et al demonstrated that TAS-116, a novel Hsp90 inhibitor, enhances radiosensitivity of human cancer cells[34]. SAHA (Suberoylanilidehydroxamic acid) is an effective radiosensitizer that probably interferes in double-strand break (DSB) repair when used in combination with ionizing radiation on glioblastoma cancer cells [35]. The PARP inhibitorAZD2281 radiosensitizes a pancreatic cell line in the presence of low and high linear energy transfer radiation [30].

Radiation induced SSBs (single strand breaks) and DSBs frequently contain 5’ hydroxyls and 3’ phosphate group that must be processed for the subsequent action of DNA polymerase and ligases [33, 36]. PNKP (polynucleotide kinase/phosphatase), the only protein which restores the 3´- hydroxyl group and 5´- phosphate group needed to seal the broken DNA has bifunctional potential: kinase and phosphatase activity [36]. It has been suggested that-, the terminal base pairs of double-stranded substrates near the 3´-phosphate are destabilized by PNKP to allow substrate access to the active site [37]. PNKP functions in multiple ionizing radiation induced repair pathways like base excision repair (BER), DNA SSB repair and non-homologous end joining (NHEJ) making it an attractive therapeutic target [38]. Thus inhibition of PNKP, an important target for enhancing the high LET radiation efficacy, may be an effective radiosensitizer.

The hPNKP inhibitor, A12B4C3 is reported to inhibit mammalian PNKP activity. It disrupts the secondary structure of PNKP and acts as a noncompetitive inhibitor that allosterically regulates the phosphatase activity of human PNKP [33]. A12B4C3 treatment was shown to radiosensitizes human A549 lung carcinoma, MDA-MB-231 human breast cancer cells and human myeloid leukemia cells with γ-radiation but has not been studied in a radioresistant cells with C-ion irradiation [34, 38].

Hence the present study is targeting PNKP inhibition in combination with C-ion radiation to enhance the cell killing in radioresistant prostate cancer cell line PC-3.PNKP inhibition may be a promising approach to enhance clinical outcome of C-ion radiation in radioresistant cancer cells.

## Methods

### Cell culture

PC-3 cells were obtained from National repository NCCS (National Centre for Cell Science, Pune, India) and maintained in nutrient mixture F-12 Ham, K medium supplemented with 10% fetal bovine serum in presence of antibiotic solution (all three from Himedia, India) at 5% CO_2_ in a humidified atmosphere at 37°C. Cultured cells were irradiated at70-80% confluency.

### Charged particle radiation

For irradiation of cells, the 15 UD Pelletron acceleratorat Inter University Acclerator Centre (IUAC), New Delhi, India,was utilized. Cells were maintained in Radiation Biology Laboratory at IUAC, New Delhi. The irradiation of cells was done using heavy ion irradiation facility ASPIRE, where the dosimetry is done using silicon surface barrier detectors [39, 40]. We have used ^12^C beam with 85MeV (equivalent to 7.08 MeV/nucleon) energy from the accelerator. The energy of the beam on the cell surface was 62 MeV (equivalent 5.16 MeV/nucleon) with entrance LET 287 kev/μm as calculated by SRIM software [41]. The beam flux was maintained at about 2 x 10^5^particles/sq.cm/sec. The dose in Gy was calculated using the following standard relation, where the cell is taken to be water equivalent.

$$Dose(Gy)=\;1.6*\;10^{-9}*LET(keV÷\mu m)*Fluence(particles÷sq.cm)$$

[42]

### Treatment

**Fig 1:**
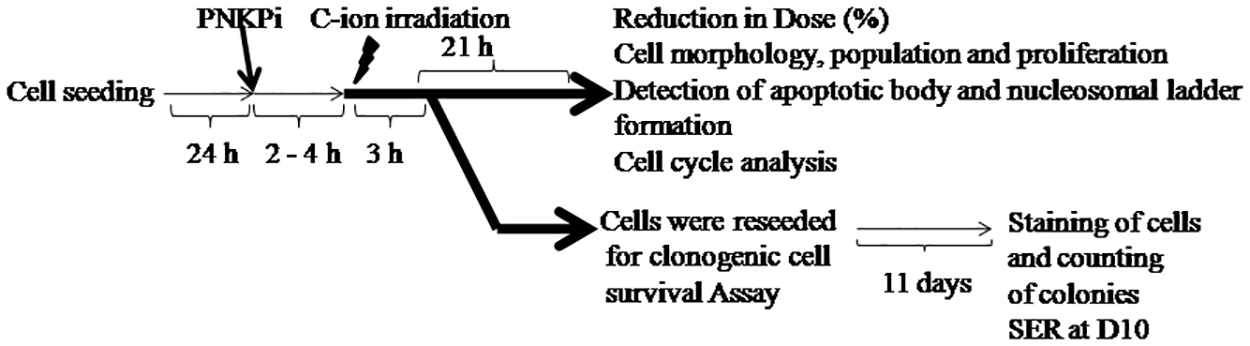
Schematic work flow for experiments.

0.5×10^5^ cells/plate on 35 mm petri dishes were seeded 24hr prior to irradiation and treated with the PNKP inhibitor (PNKPi) A12B4C3 (Sigma Aldrich, USA). A12B4C3 stock solution was made in dimethyl sulfoxide (DMSO) and serial dilutions were prepared in culture medium. The control vehicle was culture media containing amounts of DMSO equivalent to those present in PNKPi. Cells were treated with PNKPi 2–4 hr before irradiation “Fig 1”.

### Clonogenic cell survival

For clonogenic cell survival assay, cells were seeded 24 hr before irradiation in 35mm Nunc culture dishes (Sigma-Aldrich USA). Thereafter cells were irradiated and 3 hr after irridiation, cells were counted by Countess^™^ automated cell counter (Invitrogen, USA) through trypan blue staining and then 200 to 2000 cells, depending upon the dose (larger the dose of radiation, lower the plating efficiency therfore more cells are seeded to obtain the desired colony count) were reseeded in 60mm Tarsons culture dishes. Treatment was done with PNKPi 2–4 hr before irradiation at different concentration. After replating, cells were incubated for 11 days to permit cells to either undergo death by several mechanism (i.e. apoptosis, mitotic catastrophe) or to survive and form colonies.Cell survival was assessed by the standard colony formation assay as described [25].

The clonogenic survival curve was fitted to Linear Quadratic model given by the formula:

SF=e[-αD-βD^2]

In which α and β are radiation sensitivity parameters and D is the dose [28, 43]. Each point in the corresponding graphs represents the mean surviving fraction calculated from four independent experiments, and error bars represent the standard deviation (SD).

### The sensitizer enhancement ratio (SER) and reduction in dose (%)

The SER is an indicator of the radiosensitizing effect of a drug of interest and it was calculated as the ratio of the doses at 10% survival level in the absence of drug to that in the presence of drug [44, 45].

$$\text{SER}={\text{D}}_{\text{10}}\;\text{value}\;\text{of}\;\text{radiation}/{\text{D}}_{\text{10}}\;\text{value}\;\text{of}\;\text{radiation}\;\text{with}\;\text{drug}$$

Reduction in dose (%) was calculated by following formula:

$$\text{Reduction}\;\text{in}\;\text{Dose}\;\left(\%\right)={\;\text{D}}_{10}\left(\text{with}\;\text{radiation}\right)-{\text{D}}_{10}\left(\text{with}\;\text{radiation}\;\text{and}\;\text{drug}\right)/{\text{D}}_{10}\left(\text{with}\;\text{radiation}\right)$$

### Cell morphology and cell proliferation

Cell morphology was observed under Axio Vision 4.8.2 of Carl Zeiss Fluorescence Microscope.Cell numbers were counted through Countess^™^ automated cell counter (Invitrogen, USA) using trypan blue staining. Cell proliferation was measured as described [46]. The absorbance was measured at a wavelength of 490 nm using VICTOR^™^*X*5 multilabel Plate Reader (PerkinElmer).

### Apoptotic body formation and nucleosomal ladder

Apoptotic body formation and nucleosomal ladder formation was examined after C-ion irradiation (0.5-4Gy) followed by 24hr incubation. For both experiment Ghorai et al (2015) protocol was followed [47].

### Caspase 3 activation

The activity of caspase-3 was determined by colorimetric caspase-3 (Sigma) assay with minor modifications [48]. In short, after irradiation cells were grown for further 24 hr after trypsinization, cells were washed with cold PBS, and pelleted down. Then cells were lysed in 50*μ*L of chilled RIPA buffer (Sigma Aldrich, USA) on ice for 30 min. The lysate was centrifuged at 14,000 rpm for 15min at 4°C to precipitate cell debris and the supernatant was collected. The total 100*μ*l reaction mixture contained 30*μ*l cell lysate, caspase-3 substrateacetyl-Asp-Glu-Val-Asp-*p*-nitroanilide (final concentration200μM) and 50mM sodium phosphate buffer (pH 7) in a 96-well plate. Reaction mixture was incubated for 90 mins at 37°C water bath. Absorbance was measured in spectrophotometer at 405 nm.

### Cell cycle distribution by FACS

Propidium Iodide (PI) staining was used to analyze the phases of cell cycle as described [49]. In short, after irradiation (0.5-4Gy of C-ion), cells were incubated for 24 hr and then collected in medium by scraping, rinsed with PBS, centrifuged, put in 70% precooled ethanol, fixed at 4°C overnight. After fixation, cells were washed thrice with cold PBS and then stained with staining solution (10μg/ml Propidium Iodide, 100μg/ml of DNase-free RNase and 0.1% v/v triton-100) and incubated at 37°C for 15 min. The phases of cell cycle were detected on the BD Accuri^™^ Flow Cytometer (B.D. Biosciences, USA) and data analyzed by BD Accuri C6 software.

### Statistical analysis

In this study data were averaged from at least three biological repeats and presented as mean ± standard deviation wherever required. Different samples means were compared by one-way analysis of variance (ANOVA) followed by Newman–Keuls multiple comparison tests. P<0.05 was considered significant. All statistical analysis was done using Prism ver. 5 (GraphPad Software Inc., USA).

## Result

### Radiosensitizing effect of PNKPi in PC-3 cells after exposure of C-ion beam

**Fig 2:**
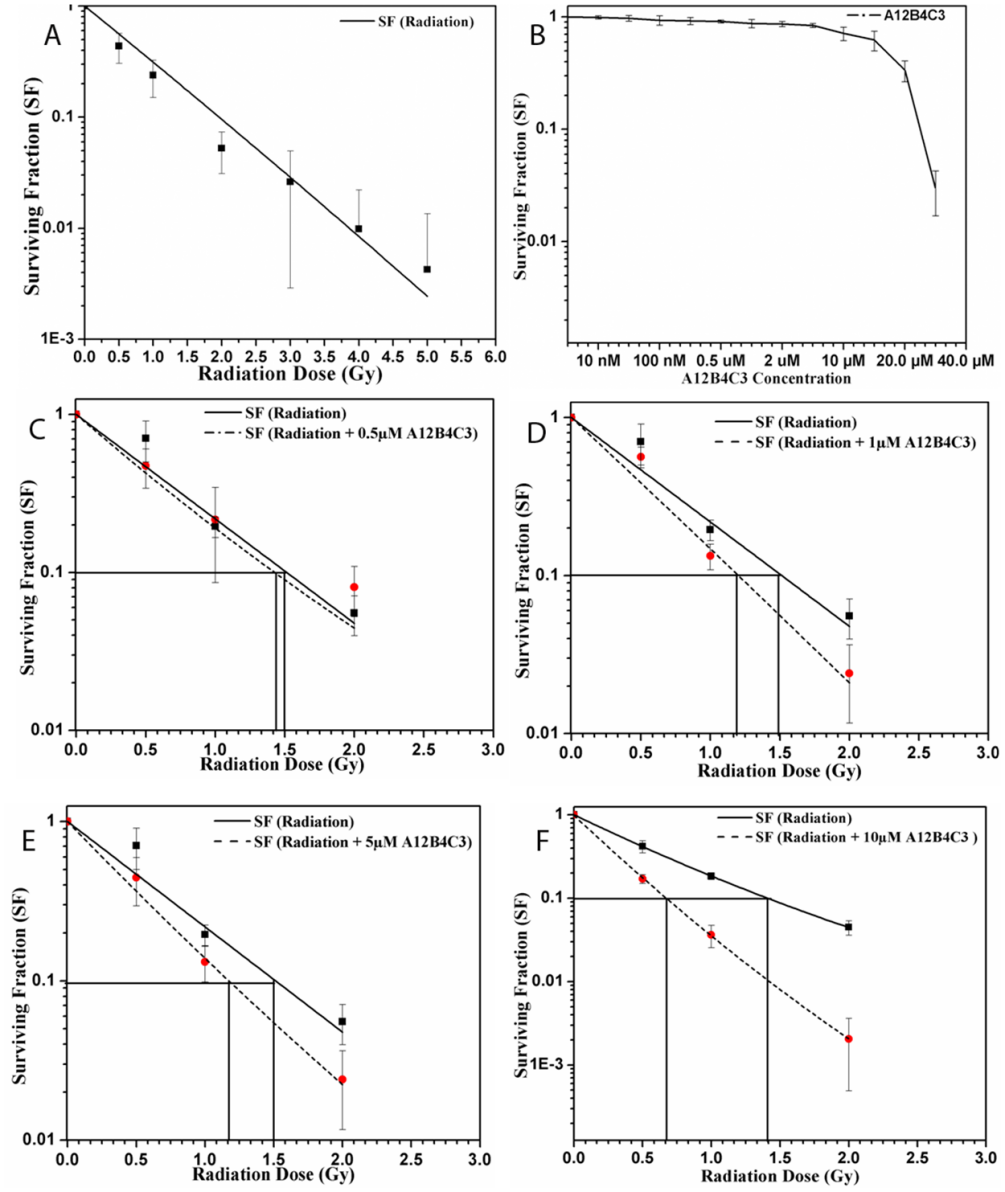
Surviving fraction (SF) graphs of PC-3 cells. (**A)**Note a dose dependent SF curve when PC-3 cell were treated with ^12^C beam only. **(B)** Effect of different concentration of PNKPi A12B4C3 on SF of PC-3 cells note that cells were least toxic till 10μM concentration.**(C-F)** Effect of different doses of ^12^C beam (**―**)with different concentration of A12B4C3 (…..) note a decrease in the SF as the concentration of A12B4C3 increases with ^12^C irradiation.

The radiation dose response of the C-ion beam and the cytotoxicity of PNKPi in PC-3 cells were determined by clonogenic cell survival assay. As the dose of C-ion beam increases (0.5Gy—5Gy) surviving fraction of PC-3 cells decreases “Fig 2A”. Surviving fraction of PC-3 cells when treated with only PNKPi (A12B4C3) showed little toxicityfor up to a 10μM concentration “Fig 2B”. For evaluation of any synergistic effect that could moderate significant cell death three dose of C-ion beams (0.5Gy, 1Gy, or 2Gy) and four concentration of PNKPi (0.5μM, 1μM, 5μM or 10μM) were used to check the clonogenic cell survival. Surviving fraction for combined treatments were calculated and normalized to a drug control sample, i.e. treated with only respective concentration of PNKPi in DMSO. The normalized surviving fractions were then compared with only C-ion irradiated samples. In terms of cytoxicity, additive effects were seen for combination treatment“Fig 2C–2F”.

### The SER at D_10_ and reduction in dose (%)

**Fig 3:**
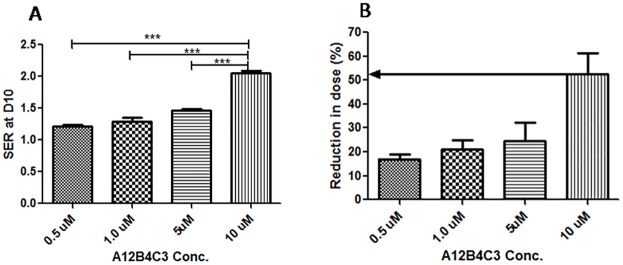
**(A)**Sensitizer Enhacement Ratio (SER) at D10 (dose giving a survival of 10%) in PC-3 cells irradiated by ^12^C beam. D_10_ plots are shown in Fig 1C–1F. **(B)** represents reduction in dose (%). >50% of the dose of irradiation got reduced by using 10μM concentration of A12B4C3 with ^12^C irradiation.***p<0.001,Significance of difference from 10μM concentration of A12B4C3.

SER values were calculated at D_10_ (dose at 10% survival level)and it ranged from 1.4 to 2.1. 10μM PNKPi had maximum SER ratio when compared with rest of the PNKPi concentrations“Fig 3A”. As illustrated by “Fig 3B”, 10 μM PNKPi reduced thesurvival by more than50%. Therefore, for rest of the combination treatment experiment, 2Gy or 4Gy of C-ion radiation were combined with 10μM PNKPi.

### Effects on cell morphology, population and proliferation

**Fig 4:**
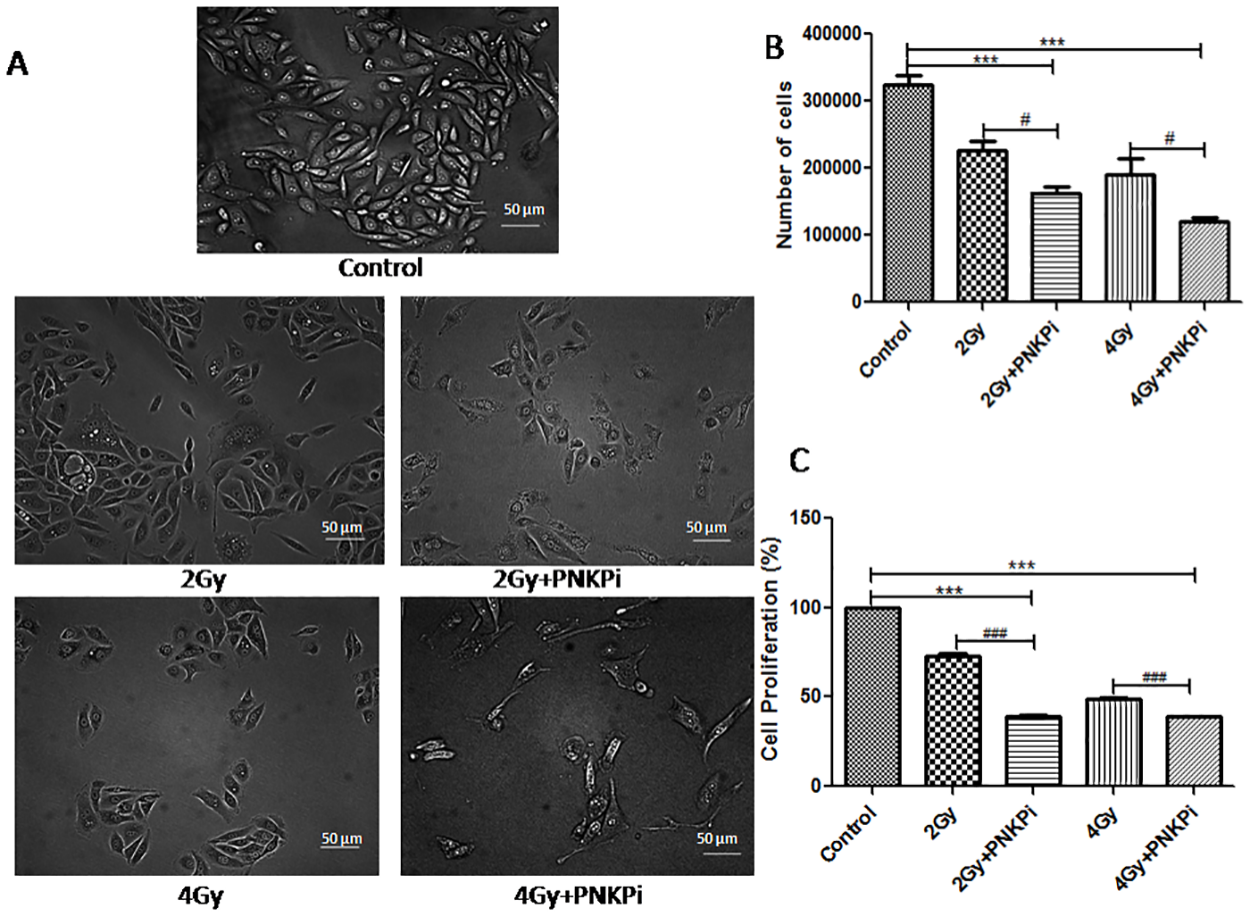
**(A)** Morphological changes of PC-3 cells with and without PNKPi (A12B4C3) treatment after 2Gy and 4Gy of ^12^C beam. The combination treated group showedmorphological changes as compare to only irradiated and control cells. **(B)**Cellpopulation decreased in combination treatment samples.***p<0.001, significance of difference from control and ^#^p<0.05in comparison with the only irradiated group.**(C)**Cell proliferaton (%) decreased when ^12^C beam is combined with PNKPi. ***p<0.001, significance of difference from control and ^###^p<0.001, significance of difference from only irradiated group respectively.

Morphology of cells was altered and cells did not show normal apperance when the C-ion beam was combined with PNKPi “Fig 4A”. The population of cells in combination treatment was reduced to 0.73±0.08, 0.65±0.10 fold as compared to the surviving fractions for 2Gy and 4Gy alone. The number of cells was reduced to 0.70±0.06, 0.50±0.01, 0.59±0.01, 0.37±0.02 in 2Gy, 2Gy+PNKPi, 4Gy and 4Gy+PNKPi treated cells as compared to control samples “Fig 4B”. Cell proliferation data showed that viabilty of cellsin combination with PNKPi decreased to 73.04±1.42%, 39.2±0.28%, 49.08±0.27%, 38.8±0.16% in the 2Gy, 2Gy+PNKPi, 4Gy and 4Gy+PNKPi group as compared to control (100%) and 53.75±0.71%, 79.08±0.77% in the 2Gy+PNKPi and 4Gy+PNKPi groups as compared to cells that were irradiated only i.e. 2Gy and 4Gy respectively “Fig 4C”.

### Combined treatment of C-ion beam and PNKPi stimulate cell death through apoptotic body and nucleosomal DNA ladder formation

**Fig 5:**
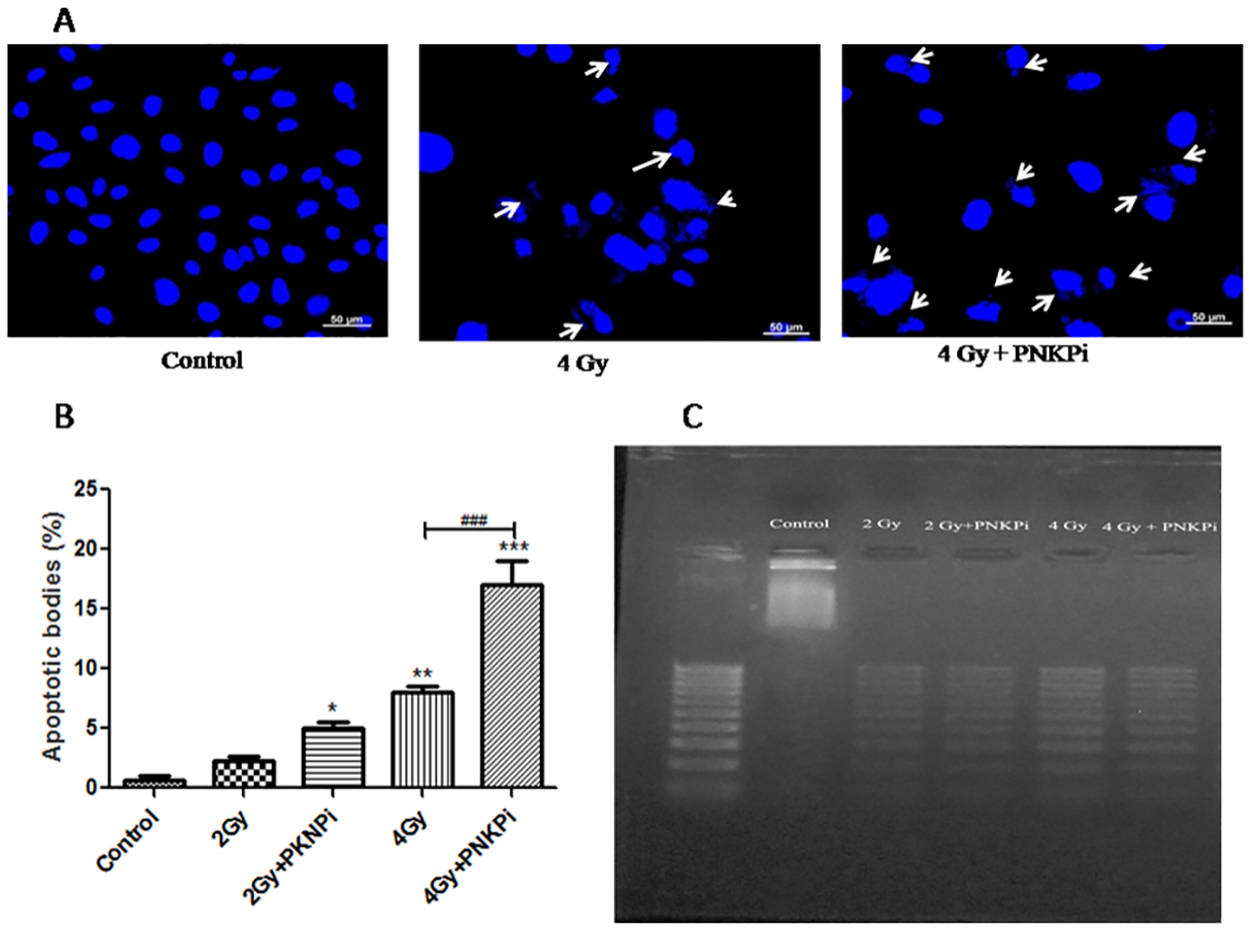
**(A)** Morphology of apoptotic body formation by ^12^C irradiation with and without PNKPi after 24 hr were shown. Apoptotic bodies are shown by ‘*arrow sign*’. Scale bar represents 50μm. **(B)** % apoptotic bodies were counted randomly taken at least 150 cells at each dose group. *Bar diagram* showing apoptotic body (%). *p<0.05, **p<0.01, ***p<0.001; significance of difference in comparison from control, ^###^p<0.001, significance of difference compare to the only irradiated group. **(C)** Gel image of nucleosomal ladder assay formation after staining with ehidium bromide. Distinct ladders were formed when PC-3 cells were treated with ^12^C beam in the presence of PNKPi compare to control.

There was a significantly higher number (2.12±0.57, 2.14±1.7 fold higher) of apoptotic bodies in cells which were treated with PNKPi in combination with C-ion beam as compared to only irradiated cells, i.e. 2 Gy and 4 Gy. We observed 3.5±1.0, 7.5±1.7, 12±1.7 and 25.5±6.24 fold increases in the number of apoptotic bodies in 2Gy, 2Gy+PNKPi, 4Gy and 4Gy+PNKPi treated cells as compared to control cells respectively “Fig 5A and 5B”.

A clear and more distinct ladder were formed when irradiation dose (2Gy and 4Gy) is combined with PNKPi, as compared to control cells “Fig 5C”.

### Caspase 3 activation

**Fig 6:**
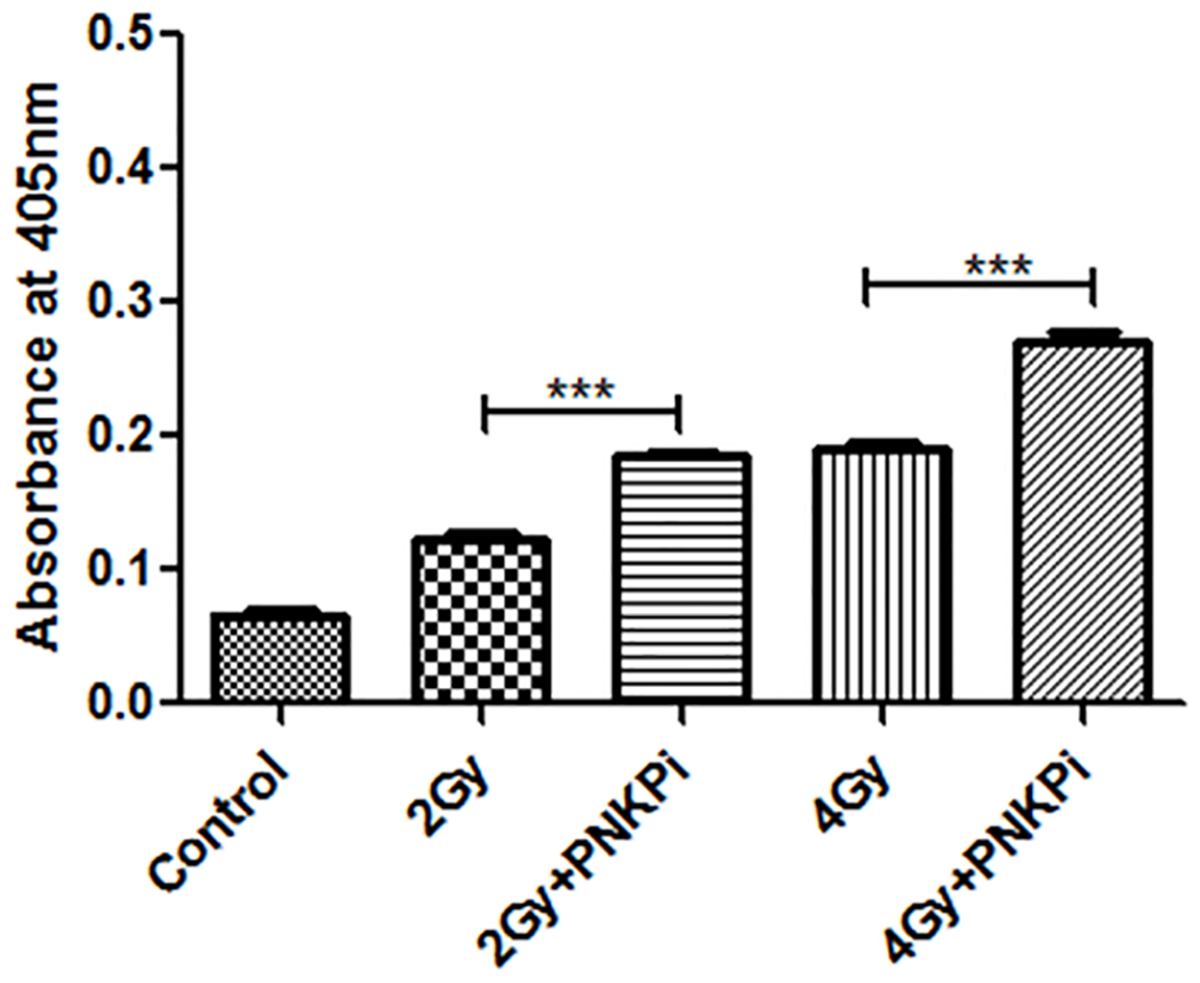
Bar graph represents the increase in absorbance (OD) at 405 nm due to the release of *p*-nitroanilide. Caspase-3 activity was highly increased in PNKPi treated group compare to only irradiated group.***p<0.001 significance of difference from only irradiated group.

There was an enhancement (1.49±0.89 and 1.41±0.83 fold increase) in the activity of caspase 3 when PC-3 cells were exposed to a C-ion beam in the presence of PNKPi as compared to cells that were only irradiated, i. e.2Gy and 4Gy respectively. We found 1.86±1.3, 2.78±0.89, 2.87±1.08 and 4.07±2.9 fold increases in 2Gy, 2Gy+PNKPi, 4Gy and 4Gy+PNKPi treated cells respectively in comparison with control cells “Fig 6”.

### Carbon ion irradiation induces significant G2/M arrest when combined with PNKPi

**Fig 7:**
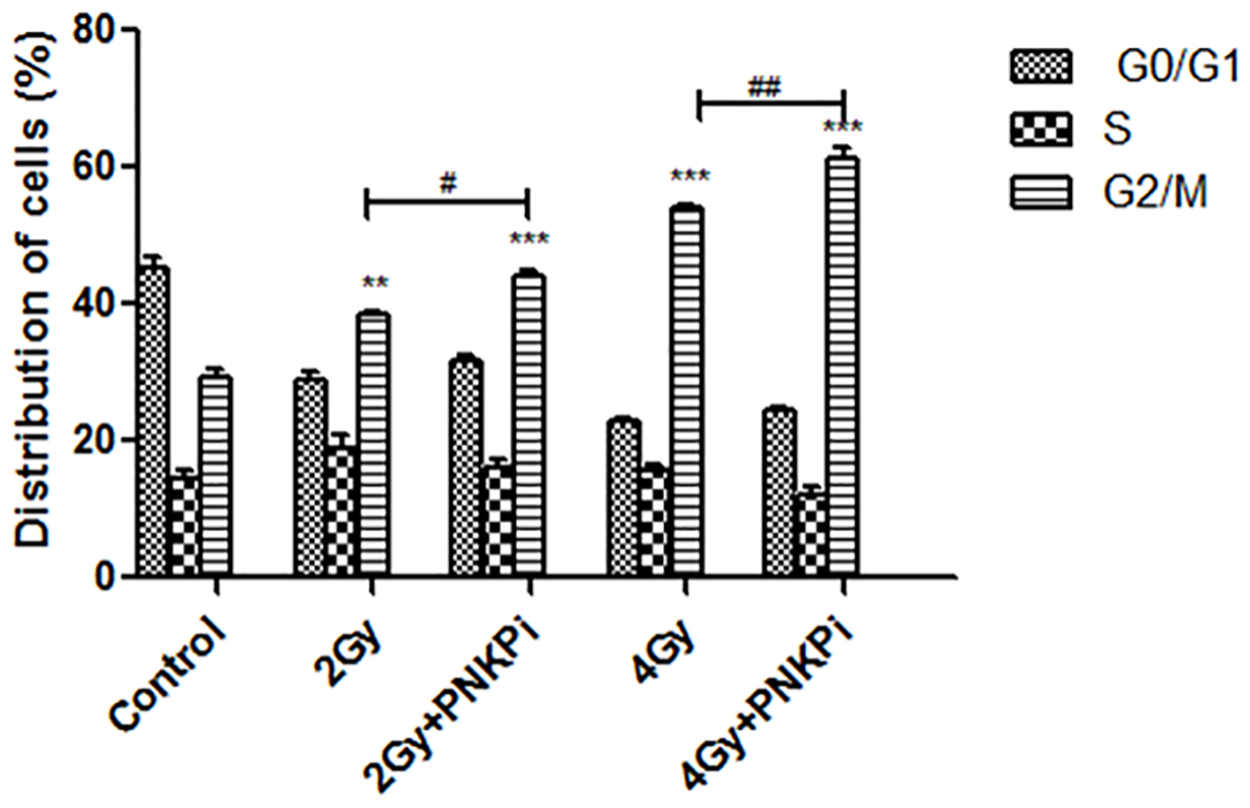
FACS analysis showing distribution of cells % (cell cycle arrest at different phases). G2/M phase arrest was enhanced in PNKPi treated group comapred to control cells. **p<0.01; ***p<0.001, significance of difference compared to control and ^#^p<0.05, ^##^p<0.01 in comparison with the only irradiated group.

Cell cycle arrest at G2/M phase was enhanced by 1.14±0.45, 1.13±0.5 fold in the presence of PNKPi as compared to cells given radiation alone, i.e. 2 Gy and 4 Gy respectively. Moreover, we observed 1.31±0.45, 1.50±0.90, 1.84±0.45, 2.09±1.31 fold increase at G2/M phase in 2Gy, 2Gy+PNKPi, 4Gy and 4Gy+PNKPi treated cells as compared to control cells respectively “Fig 7”.

## Discussion

An approach to overcome the radioresistance problem and to achieve a reduction in the cost of carbon ion radiotherapy is still under investigation. However, effective radiosensitizers like PARP inhibitors or Protein Kinase (PK) inhibitors,which may promote radiation-induced senescence,are currently at various stages of the drug development process [31,32]. Here we have tried to reduce the radiation dose required by using aradiosensitizer (PNKPi) and targeting radiodioresistance problem by selecting a radioresistant prostate cancer PC-3 cells simultaneously. Keeping these in mind, the combined effect of C-ion radiation and PNKPi on PC-3 cells has been tested in the present study. In order to know the cell killing effect of C-ion radiation, we first identified a dose that generates10% cell survival in PC-3 cells. Surviving Fraction (SF) graphs “Fig 2A” revealed that PC-3 cells show cell death in dose dependent manner when exposed to the C-ion radiation alone. Next, we tried to find out a concentration which exhibit less cytotoxic effect and has significant radiosensitizing effect when combined with C-ion radiation. Previous report suggest cytotoxicity of A12B4C3 at 100μmols/L in A549 and MDA-MB-231cells [50]and at 25 μmols/L in AML-5 cells [36]. In our study, we have found that10μM A12B4C3 did not showsignificant toxicity in PC-3 cells “Fig 2B”. Further, MTT assay showed that there was no cytotoxicity of A12B4C3 up to 100μM. To evaluate the synergistic effect of C-ion radiation and PNKPi we have choosen four different concentartion of A12B4C3 (0.5μM, 1μM, 5μM and 10μM) with three different doses of C-ion irradiation (0.5Gy, 2Gy and 4Gy) for clonogenic cell survival assay and found that C-ion irradiation causes enhancement in cell death when pretreated with PNKPi“Figs 2C–2F”. We further observed that 10μM concentration of A12B4C3 showed a significant sensitizer enhancement ratio (SER) at D_10_ (dose at 10% survival level)“Fig 3A”. These results are in agreement with the works by Zereshkian et al [36]. Moreover, by using A12B4C3, the effective radiation dose was reduced by more than 50% “Fig 3B”.

Therefore we selected 10μM dose of A12B4C3 for the rest of the experiments, which is less than the dose reported of A12B4C3 in other studies [36,50]. This synergistic action also affected cell mophology and population parameters. A reduction in the cell population was observed in dose dependent manner after C-ion beam exposure, which was further reduced by 0.73±0.08, 0.65±0.10 fold by incorporation of PNKPi “Fig 4B”. Statistically, cell proliferation was also reduced significantly to 73.04±1.42%,49.08±0.27% in the radiation alone group (2Gy, 4Gy) and 39.2±0.28%, 38.8±0.16% by radiation plus drug treatment group (2Gy +PNKPi, 4Gy+PNKPi) respectively as compared to control (100%)“Fig 4B”.

Cell death mode induction through C-ion radiation is still unknown. Many reports suggested that it is through apoptosis and independent of p53 status [15]. PC-3 cell line is null for p53. Our findings based on DAPI images and ladder assay data showed a clear picture of fragmented nuclei and nucleosomal ladder formation that predicts PC-3 cells are undergoing p53-independent apoptosis“Figs 5A–5C”. To confirm apoptosis, we further measured the activity of caspase 3. We found 1.86±1.30, 2.87±1.08, 2.78±0.89, and 4.07±2.9 fold enhancement in caspase 3 activity when PC-3 cells treated with PNKPi in combination with C-ion beam in radiation alone group (2Gy, 4Gy) and by radiation plus drug treatment group (2Gy+PNKPi, 4Gy+PNKPi) respectively as compared to control “Fig 6”. Some studies suggest that radiation induces cell cycle arrest at G2/M phase [48,49]. Our study also observed G2/M phase arrest after C-ion irradiation. We further report that in combination with A12B4C3, the level of G2/M phase arrest was enhanced by 1.31±0.45, 1.84±0.45, 1.50±0.90, and 2.09±1.31fold in radiation alone group (2 Gy, 4Gy) and by radiation plus drug treatment group,(2Gy+PNKPi, 4 Gy+PNKPi) respectively as compared to control “Fig 7”.

## Conclusion

Present findngs led us to conclude that, PNKP inhibition through A12B4C3 may be a suitable alternative to enhance cell death in radioresistant prostate cancer cell line like PC-3 on the exposure to carbon ion radiation. To the best of our knowledge, this is the first study where it has been clearly demonstrated that,the efficiency of cell killing by carbon ion (^12^C ion) irradiation gets enhanced by the pretreatment of PNKPi (A12B4C3) in the cell line PC-3. It is also suggested that, combined treatment of C-ion radiationin 2 Gy-4 Gy range with a low concentration of the A12B4C3 radiosensitizer may be an effective method to kill cancer cells and to stop their recurrence. However, further studies involving i) understanding of molecular mechanism(s)behind cell death like apoptosis/senescence/autophagy ii) PI3K/Akt/mTOR signaling pathwayand iii) *In Vivo* experiments to evaluate this candidate therapy iv) use of a normal cell line to compare effects of PNKP inhibition on radio-sensitization of normal tissues will be investigated next to provide a cleare picture behind the radiosensitization effect of PNKPi.
